# Diffusion dynamics of synaptic molecules during inhibitory postsynaptic plasticity

**DOI:** 10.3389/fncel.2014.00300

**Published:** 2014-09-23

**Authors:** Enrica Maria Petrini, Andrea Barberis

**Affiliations:** Department of Neuroscience and Brain Technologies, Istituto Italiano di TecnologiaGenoa, Italy

**Keywords:** GABA_A_ receptors, GABAergic plasticity, scaffold proteins, single particle tracking, gephyrin, lateral diffusion, intracellular trafficking, phosphorylation

## Abstract

The plasticity of inhibitory transmission is expected to play a key role in the modulation of neuronal excitability and network function. Over the last two decades, the investigation of the determinants of inhibitory synaptic plasticity has allowed distinguishing presynaptic and postsynaptic mechanisms. While there has been a remarkable progress in the characterization of presynaptically-expressed plasticity of inhibition, the postsynaptic mechanisms of inhibitory long-term synaptic plasticity only begin to be unraveled. At postsynaptic level, the expression of inhibitory synaptic plasticity involves the rearrangement of the postsynaptic molecular components of the GABAergic synapse, including GABA_A_ receptors, scaffold proteins and structural molecules. This implies a dynamic modulation of receptor intracellular trafficking and receptor surface lateral diffusion, along with regulation of the availability and distribution of scaffold proteins. This Review will focus on the mechanisms of the multifaceted molecular reorganization of the inhibitory synapse during postsynaptic plasticity, with special emphasis on the key role of protein dynamics to ensure prompt and reliable activity-dependent adjustments of synaptic strength.

## Introduction

γ-aminobutyric acid (GABA) receptors mediate the majority of inhibitory signals in the brain. GABAergic inhibition consists of a fast and precisely timed component generated by the vesicular release of GABA in the synaptic cleft (phasic inhibition), and of a persistent tonic conductance due to receptor activation by ambient GABA (tonic inhibition) (Farrant and Nusser, 2005; Olsen and Sieghart, 2008). While phasic inhibitory transmission is mainly mediated by the activation of α1-3β2-3γ GABA_A_ receptors (GABA_A_Rs) clustered at synapses, tonic conductance arises from extrasynaptic GABA_A_Rs typically composed of α1/4/6βδ and α5βγ. It is well known that inhibitory transmission is crucial to tune neuronal excitability and to regulate network integration. In particular, at network level, inhibitory synaptic signals are fundamental for generating coherent oscillations and selection of cell assemblies (Bartos et al., 2007; Klausberger and Somogyi, 2008; Royer et al., 2012). As such, GABAergic inhibition controls higher cognitive functions in the brain and lies at the basis of some neurological disorders when impaired (Lewis et al., 2012; Katona et al., 2014). Over the last decades, accumulating evidence has revealed that both phasic and tonic inhibitory signals can be plastic, thus raising additional possibilities for the modulation of network activity and neuronal circuit refinement (Kano et al., 1992; Saliba et al., 2012; Bright and Smart, 2013). The emerging role of inhibitory synaptic plasticity in higher brain functions has provided a strong drive towards the investigation of the underlying cellular, structural and molecular determinants (Frotscher et al., 1990; Kozhedub and Knipst, 1997). As a consequence, a variety of “inhibitory plasticities” can be identified in different brain regions such as cerebellum, hippocampus, visual cortex, ventral tegmental area, lateral amygdala (Kano et al., 1992; Marsicano et al., 2002; Patenaude et al., 2003; Maffei et al., 2006; Nugent et al., 2007; Heifets et al., 2008). However, a comprehensive knowledge of the mechanisms that lead to activity-dependent changes of inhibitory synaptic strength has been limited by the strong diversity of (i) inhibitory interneuron cell types; (ii) inhibitory synapses along the dendritic arbor and the soma; and (iii) GABA_A_ receptor subtypes. Some of the best characterized forms of inhibitory synaptic plasticity depend on changes in presynaptic GABA release (McBain and Kauer, 2009; Castillo et al., 2011). Messengers such as endocannabinoids, BDNF or NO, released from the postsynaptic cell in activity-dependent manner, retrogradely diffuse to the presynaptic terminal, where they modulate the amount of GABA released in the cleft, thereby inducing the depression or potentiation of inhibitory synaptic strength (Nugent et al., 2007; Heifets et al., 2008; Sivakumaran et al., 2009). Other forms of inhibitory synaptic plasticity are expressed postsynaptically with persistent modifications of the abundance (Nusser et al., 1998; Kilman et al., 2002; Marsden et al., 2007; Kurotani et al., 2008; Bannai et al., 2009; Muir et al., 2010; Niwa et al., 2012; Saliba et al., 2012; Nahmani and Turrigiano, 2014; Petrini et al., 2014), assortment (Houston et al., 2008; Rajalu et al., 2009) and gating (Moss et al., 1995; Jones and Westbrook, 1997; Houston et al., 2008) of postsynaptic GABA_A_ receptors. Such changes depend on a coordinated sequence of dynamic events that tune receptor delivery to, stabilization at, and removal from synapses. Additionally, at postsynaptic level, activity-dependent changes of the chloride transporters can affect inhibitory synaptic currents by altering the postsynaptic chloride driving force (Rivera et al., 1999; Sun and Murali, 1999). Moreover, intracellular chloride has been reported to act as a biochemical messenger by influencing the expression of different GABA_A_R subtypes (Succol et al., 2012). Overall, postsynaptic forms of inhibitory synaptic plasticity rely on complex processes leading to the active reorganization of GABAergic synapses at molecular level. Of note, some of the determinants of postsynaptic inhibitory plasticity are shared with excitatory synapses, suggesting that evolutionarily conserved mechanisms adjust synaptic strength through the coordinated control of postsynaptic receptor availability.

This Review will focus on the key role of molecule dynamics in the expression of inhibitory postsynaptic plasticity, with special emphasis on the activity-regulated changes in receptor trafficking, receptor lateral mobility and scaffold protein dynamic organization. Moreover, some aspects of the postsynaptic plasticity of excitatory and inhibitory synapses will be compared in order to highlight convergent points in the regulation of cell excitability.

## Role of GABA_A_ receptor intracellular trafficking in the expression of inhibitory synaptic plasticity

The initial demonstrations that GABA_A_Rs are brought to the neuronal surface by exocytosis and removed by clathrin-mediated endocytosis (Tehrani and Barnes, 1993; Kittler et al., 2000) represent the first indications that neurotransmitter receptors are not fixed at the neuronal membrane but exchange between surface and intracellular compartments. Since then, many laboratories have contributed to elucidate the multiple steps of GABA_A_Rs intracellular trafficking and have extended their studies to other neurotransmitter receptors (Maloteaux and Hermans, 1994; Luscher et al., 1999; Kittler et al., 2000; Park et al., 2004; Bogdanov et al., 2006). The large number of proteins that assist GABA_A_R intracellular dynamics (namely their exocytic/endocytic pathways and their sorting to degradation or recycling) will not be addressed here, since they have been extensively discussed in excellent reviews (Chen and Olsen, 2007; Arancibia-Cárcamo and Kittler, 2009; Jacob et al., 2009; Luscher et al., 2011; Vithlani et al., 2011).

Receptor intracellular trafficking ensures receptor renewal in basal conditions (Charych et al., 2004; Kittler et al., 2004; Vithlani et al., 2011; Huganir and Nicoll, 2013); however, it also underlies many forms of synaptic plasticity at inhibitory and excitatory synapses by dynamically regulating surface receptor availability (Luscher et al., 2011; Huganir and Nicoll, 2013). At GABAergic synapses, postsynaptically-expressed potentiation of long-term potentiation inhibition (iLTP) depends on GABARAP-mediated increase of GABA_A_R exocytosis that in turn promotes receptors accumulation at the postsynaptic density (PSD), as observed in cultured neurons, in slices and *in vivo* (Nusser et al., 1998; Marsden et al., 2007; Kurotani et al., 2008; Nahmani and Turrigiano, 2014; Petrini et al., 2014). Conversely, inhibitory long-term depression (iLTD) has been correlated with reduced availability of GABA_A_Rs at synapses, although no consensus has been achieved yet whether this is due to altered GABA_A_R internalization or to receptor dispersal from the synapse (Kurotani et al., 2008; Bannai et al., 2009; Muir et al., 2010). Indeed, the cell-specific blockade of GABA_A_R endocytosis prevents the depression of inhibition in slices of the primary visual cortex (Kurotani et al., 2008), while it does not affect the expression of iLTD in hippocampal neuronal cultures (Bannai et al., 2009; Muir et al., 2010). Similarly, at excitatory synapses, a large body of evidence describes increased AMPA receptor exocytosis as one of the main mechanisms underlying several forms of long-term potentiation (LTP) and reduced availability of AMPA receptors to be causal for long-term depression (LTD) (reviewed in Huganir and Nicoll, 2013).

Another key element of receptor intracellular trafficking that contributes to the regulation of surface receptor number and that can play a role for the expression of postsynaptic plasticity is receptor endocytic sorting (Luscher et al., 2011; Vithlani et al., 2011). In fact, by determining the fate of endocytosed receptors towards receptor lysosomal degradation or recycling to the surface, receptor endocytic sorting can set the number of receptors actively involved in receptor turnover. Hence, the preferential routing of the receptors to the recycling or lysosomal pathway would sustain increased or reduced surface receptor number during synaptic potentiation or depression, respectively (Kittler et al., 2004; Arancibia-Cárcamo et al., 2009; Mabb and Ehlers, 2010). It should be noted that the delivery of recycling receptors to the surface can be faster than that of newly synthesized receptors, which has been estimated in a time range spanning from few minutes to hours, thus providing a faster regulation of surface receptor abundance (Connolly et al., 1999; Bogdanov et al., 2006; Renner et al., 2008).

The key role of receptor trafficking in the expression of synaptic plasticity, initially demonstrated *in vitro*, has been confirmed *in vivo* by experiments addressing ecitatory and inhibitory synaptic plasticity in the barrel cortex during cortical map formation and sensory experience (Lu et al., 2003; Clem and Barth, 2006), during fear-conditioning in the amygdala and in the nucleus accumbens (Chhatwal et al., 2005; Schierberl et al., 2011), in the medial prefrontal cortex induced by cocaine (Bellone and Luscher, 2006; Ghasemzadeh et al., 2011), in the visual cortex (Frenkel et al., 2006; Nahmani and Turrigiano, 2014), and in the hippocampus (Lee et al., 2003; Tretter et al., 2009).

## GABA_A_ receptor post-translational modifications and synaptic plasticity

There is compelling evidence that the phosphorylation and dephosphorylation of GABA_A_Rs are key events for the expression of inhibitory synaptic plasticity (Comenencia-Ortiz et al., 2014). In fact, the surface expression of GABA_A_Rs depends on the activity of several kinases and phosphatases (such as CaMKII, PKA, PKC, Src, Akt, calcineurin) that tightly modulate receptor intracellular trafficking by acting on specific sites of receptor intracellular domains. It has been initially demonstrated that the intracellular application of preactivated CaMKII potentiates GABAergic currents evoked with exogenous GABA pulses by increasing the phosphorylation of Ser 383 of GABA_A_ receptor β3 subunit (Houston et al., 2007). Recently, the CaMKII-mediated phosphorylation of Ser 383 on β3 subunit has been demonstrated to be an essential requirement for the LTP of inhibitory synaptic currents as it promotes the exocytosis and the postsynaptic accumulation/immobilization of GABA_A_R at synapses (Petrini et al., 2014). Along the same lines, the serine/threonine kinase Akt increases the number of surface α1 subunit-containing GABA_A_Rs by phosphorylating Ser 410 of the β2 subunit, thereby enhancing inhibitory synaptic transmission in the hippocampus *in vitro* and *in vivo* (Wang et al., 2003b). Akt-mediated larger delivery of GABA_A_Rs to the neuronal surface has also been observed in midbrain ventral tegmental area neurons following stress stimuli that activate δ opioid receptors and elicit postsynaptic potentiation of GABA_A_-mediated inhibitory currents (Margolis et al., 2011).

GABA_A_R phosphorylation can also tune inhibitory synaptic strength by regulating receptor endocytosis through the modulation of GABA_A_Rs interactions with the endocytic machinery. Indeed, the binding motifs for the endocytic adaptor protein AP2 on GABA_A_R β and γ2 subunits incorporate sites for phosphorylation by PKA, PKC, Akt and Src and dephosphorylation by protein phosphatase 1 (PP1), PP2A and calcineurin (Brandon et al., 2002; Wang et al., 2003a; Jovanovic et al., 2004; Kittler et al., 2008). Therefore, the phosphorylation of the these residues (or of adjacent ones) precludes the binding of AP2 to the receptors, resulting in a negative regulation of GABA_A_R endocytosis (Kittler et al., 2000, 2008; Herring et al., 2003), with the consequent increase of surface GABA_A_R number (Kittler et al., 2008; Jacob et al., 2009; Smith et al., 2012). Conversely, the dephosphorylation of the residues involved in GABA_A_R-AP2 interaction favors GABA_A_R internalization and decreases the abundance of GABA_A_Rs in the neuronal membrane. For instance, in the medial prefrontal cortex, the reduced GABAergic inhibition observed after cocaine withdrawal is the result of increased PP2A activity that promotes the dephosphorylation of Ser 408/409 on GABA_A_R β3 subunit and enhances receptor internalization (Jovanovic et al., 2004; Lu et al., 2010). In the CA1 region of the hippocampus, it has been proposed that tetanus-induced LTD of unitary IPSCs might rely on a reduction of functional GABA_A_R number at synapses following the dephosphorylation of γ2 subunit by calcineurin (Wang et al., 2003a). Recently, GABAergic plasticity observed after *in vivo* Ethanol (EtOH) administration (Liang et al., 2007; Olsen and Spigelman, 2012) has been explained by promoted α4βδ GABA_A_R endocytosis due to the interaction of GABA_A_R δ subunit with the AP2 machinery (Gonzalez et al., 2012).

Remarkably, the phosphorylation of GABA_A_Rs has been also involved in the plasticity of tonic inhibition, similarly to the plasticity of synaptic inhibition. In fact, also the abundance of surface extrasynaptic receptors has been related to phosphorylation-dependent modulation of GABA_A_R trafficking. For instance, the CaMKII-dependent phosphorylation of Ser 383 on β3 subunit, elicited by the activation of L-type voltage-gated Ca^2+^ channels, favors the exocytosis of α5-containing GABA_A_Rs, thus enhancing the non-synaptic component of GABAergic inhibition in cultured hippocampal neurons (Saliba et al., 2012). Analogously, the activation of PKC induces the phosphorylation of Ser 443 on α4 subunit and of Ser 408/409 on β3 subunit of GABA_A_R, leading to the promoted surface delivery and increased membrane stability of α4-containing receptors (Abramian et al., 2010). More recently, this mechanism has been implicated in the increase of extrasynaptic α4-containing GABA_A_Rs underlying the potentiation of tonic inhibition induced by neurosteroids (Abramian et al., 2014). Overall, the experimental evidence described above define a common rule for the plasticity of phasic and tonic inhibition, namely that the phosphorylation stabilizes and dephosphorylation destabilizes GABA_A_Rs at the neuronal surface. However, the phosphorylation of GABA_A_R has also been lately demonstrated to promote GABA_A_Rs endocytosis, thus oppositely contributing to the plasticity of tonic inhibition. Namely, the phosphorylation of Ser 410 on β2 subunit by PKC reduces the surface expression of extrasynaptic α4β2δ GABA_A_Rs, resulting in the depression of tonic inhibition in the dentate gyrus region of the hippocampus and in the thalamus (Bright and Smart, 2013). It is possible that the assortment of the α4 subunit with the β2 or β3 subunit might differentially regulate the phosphorylation and trafficking of α4-containing GABA_A_Rs, resulting in the PKC-mediated depression or potentiation of tonic inhibition, respectively (Bright and Smart, 2013; Abramian et al., 2014). Another evidence that specific phosphorylation of GABA_A_R subunits differentially affect the strength of inhibition depending on the GABA_A_R subunit assortment is provided by the phosphorylation of the Ser 410 on β2 subunit. If the phosphorylated β2 subunit is within a α1subunit-containing receptor, the membrane stability of GABA_A_Rs in increased, leading to the potentiation of GABAergic synaptic transmission; on the contrary, if the phosphorylated β2 subunit is assembled with the α4 subunit it mediates the depression of inhibitory tonic currents by reducing the number of surface receptor (Wang et al., 2003b; Bright and Smart, 2013). Of note, the phosphorylation of GABA_A_Rs can also tune inhibitory synaptic strength by directly affecting the microscopic gating of the GABA_A_Rs, hence providing a potential additional level of receptor modulation during synaptic plasticity (Moss et al., 1995; Jones and Westbrook, 1997; Brandon et al., 2000). Interestingly, Tretter et al. (2009) have studied the behavioral impact of inhibitory synaptic plasticity induced by phospho-regulated changes of GABA_A_R intracellular trafficking. Knock-in mice in which the principal sites of tyrosine phosphorylation within GABA_A_R γ2 subunit (Y365, Y367) have been mutated to phenylalanine have been probed for some cognitive aspects. The Y365/7F mice exhibit aberrant endocytic pathway due to the compromised GABA_A_R-AP2 interaction, leading to increased accumulation of synaptic GABA_A_R on pyramidal neurons of the CA3 region of the hippocampus. The resulting potentiated inhibitory transmission in these Y365/7F mice correlates with specific CA3 hippocampal-dependent deficits in spatial object recognition, such as the inability to discriminate between displaced and non-displaced objects, despite intact object recognition memory (Tretter et al., 2009).

Besides receptor phosphorylation, other post-translational modifications such as ubiquitination and palmitoylation have been implicated in the control of the surface expression of synaptic proteins through the regulation of their maturation/secretory pathway during basal activity and synaptic plasticity (Mabb and Ehlers, 2010; Vithlani et al., 2011; Lu and Roche, 2012). Ubiquitination consists in the covalent attachment of one or more copies of the 76-amino acid ubiquitin monomer to lysine residues of target proteins (Hurley and Stenmark, 2011). By serving as a sorting signal on protein cargo or by controlling the efficiency of the trafficking machinery, ubiquitination regulates protein transport between membrane compartments, hence playing a key role in the modulation of synaptic efficacy (Lin and Man, 2013). The contribution of activity-dependent receptor ubiquitination, specifically of polyubiquitination, in the translocation of proteins from the endoplasmic-reticulum (ER) has been mainly assessed for GABA_A_Rs. Indeed, increased GABA_A_R ubiquitination on β3 subunit upon chronic blockade of neuronal activity redirects newly assembled receptor from the ER back into the cytosol for subsequent proteasomal degradation, thus reducing GABA_A_R synaptic accumulation and decreasing synaptic inhibition (Saliba et al., 2007). Similarly, massive activation of L-type voltage-gated calcium channels (VGCCs) depresses the efficacy of inhibitory synaptic transmission by negatively controlling receptor turnover and membrane stability with the ubiquitin-proteasome system (Saliba et al., 2009). On the contrary, increased neuronal activity decreases GABA_A_R ubiquitination and enhances receptor stability in the plasma membrane (Saliba et al., 2007). Those pieces of evidence suggest that the ubiquitin-dependent proteasomal degradation is involved in a bidirectional adaptive modulation of surface receptor number. The ubiquitination can also trigger GABA_A_R degradation via the lysosomal pathway, when this post-translational modification involves a motif within the intracellular domain of the γ2 subunit (Arancibia-Cárcamo et al., 2009).

Another receptor post-translational modification is palmitoylation, a reversible lipid modification occurring at the intracellular domain of neurotransmitter receptors—such as GABA_A_R and AMPA receptors (Rathenberg et al., 2004; Hayashi et al., 2005), scaffold proteins (El-Husseini Ael et al., 2002; Dejanovic et al., 2014), and other receptor interacting proteins (Hanley and Henley, 2010). The palmitoylation of GABA_A_R γ2 subunit has been demonstrated to favor the assembly and clustering of GABA_A_Rs by promoting their translocation through the Golgi apparatus to the neuronal membrane (Keller et al., 2004; Rathenberg et al., 2004; Fang et al., 2006). Impairment of the Golgi-specific palmitoyl acyltransferase GODZ, that mediates the palmitoylation of GABA_A_R γ2 subunit, selectively reduces GABA_A_Rs at synapse, thus decreasing the amplitude of inhibitory synaptic currents (Fang et al., 2006).

## Additional mechanisms for the postsynaptic control of inhibitory synaptic strength

As introduced above, the strength of inhibitory signals can be also tuned by the intracellular chloride gradient that, in turn, depends on chloride transporters (Payne et al., 2003). It has been reported that the local decrease of K-Cl-cotransporter 2 (KCC2) efficiency reduces the strength of inhibition (Woodin et al., 2003). Even more importantly, the cotransport function is susceptible to changes in network activity. That is, increased glutamatergic transmission affects the chloride transport through the regulation of KCC2 phosphorylation at Ser 940 residue by PKC and PP1, which consecutively modulates KCC2 membrane trafficking (Rinehart et al., 2009; Lee et al., 2011). As a result of such altered chloride concentration, the activity-dependent regulation of IPSCs reversal potential directly shapes synaptic transmission (Wang et al., 2006; Saraga et al., 2008). Recently, it has also been demonstrated that the intracellular chloride concentration can determine the postsynaptic expression of GABA_A_R α1, α3 and δ subunits, resulting in altered inhibitory synaptic transmission (Succol et al., 2012). Given the importance of these GABA_A_R subunits in controlling the strength of phasic and tonic GABAergic activity, such changes in GABA_A_R subunit expression are expected to strongly impact inhibitory network functioning (Brickley et al., 1996; Mozrzymas et al., 2007; Brickley and Mody, 2012).

Gene expression and mRNA translation of receptor subunits can further modulate the surface availability and synaptic accumulation of neurotransmitter receptors during synaptic plasticity (Mameli et al., 2007; Jung et al., 2014). For instance, the upregulation of GABA_A_Rs and gephyrin proteins contributes to iLTP expression, while their downregulation has been observed during status epilepticus in the CA1 region of the hippocampus (Peng et al., 2004; González et al., 2013; Petrini et al., 2014). Moreover, it has been reported that fear conditioning regulates the gene expression of gephyrin in the amygdala (Ressler et al., 2002; Chhatwal et al., 2005). It is worth mentioning that mRNA encoding for proteins involved in the same plasticity process are co-assembled into the same RNA granules and targeted to dendrites (Gao et al., 2008). The coordination of such multiplexed dendritic targeting of different RNAs implies an adequate regulation to orchestrate gene expression at the synapse in the most metabolically and temporally efficient way.

## Diffusion trapping of receptor lateral mobility influences synaptic signaling

**Figure 1:**
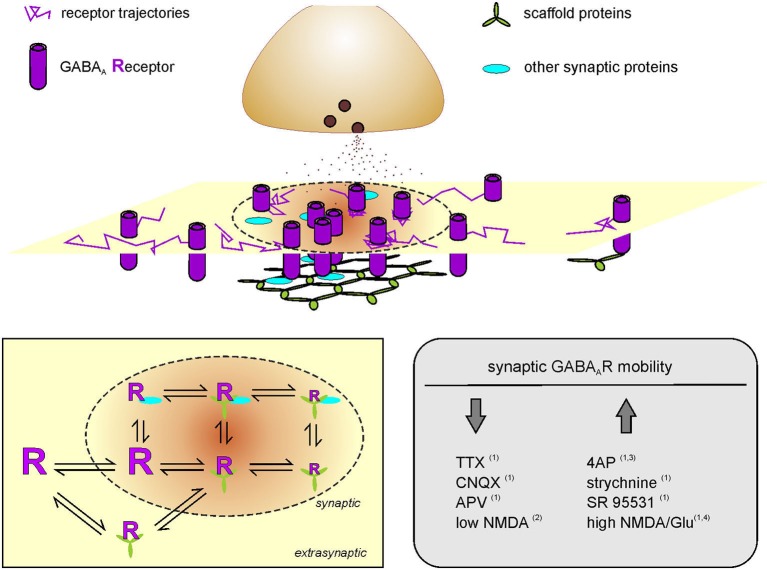
**Activity-dependent modulation of GABA_A_ receptor diffusion trapping**. Schematic representation of a postsynaptic membrane where GABA_A_Rs laterally diffuse. Receptor trajectories are more confined at the inhibitory PSD (dotted line) as compared to extrasynaptic compartments. The reduced diffusion and transient stabilization of GABA_A_Rs at the inhibitory synapse is favored by receptor interactions with scaffold proteins and by the presence of other synaptic proteins that provide molecular obstacles to receptor dynamics. Receptor scaffold interactions can also occur at extrasynaptic areas. Left inset: Diffusion-reaction model of GABA_A_R surface mobility and interaction with stabilizing proteins. GABA_A_Rs can freely diffuse in the neuronal membrane and exchange between synaptic and extrasynaptic compartments. At the inhibitory synapse, receptor interaction with scaffold proteins (green) and/or other postsynaptic proteins (cyan) can reduce and confine GABA_A_R dynamics to various levels of stabilization. Receptor mobility is represented by the size of the letter “R”. Some receptor-scaffold complexes can be formed also extrasynaptically and exchange with the synapse as a whole. Right inset right: Summary of the modulation of surface GABA_A_R mobility at inhibitory synapses in the hippocampus, upon pharmacologically-induced changes of neuronal activity. Bannai et al. (2009); Muir et al. (2010); Niwa et al. (2012) and Petrini et al. (2014).

The early 2000s have witnessed a revolution in the notion of neurotransmitter receptor trafficking, when the direct observation of individual receptor lateral diffusion in the plane of the neuronal membrane was documented (Meier et al., 2001; Borgdorff and Choquet, 2002; Tovar and Westbrook, 2002; Dahan et al., 2003; Meissner and Haberlein, 2003; Tardin et al., 2003; Thomas et al., 2005; Burli et al., 2010; Fernandes et al., 2010). As such, the concept of the synapse has turned from a static entity endowed with semi-permanent neurotransmitter receptors to a dynamic structure where the number, type and position of receptors constantly change by lateral diffusion. The lateral mobility of surface neurotransmitter receptors in the neuronal membrane consists of thermally-driven Brownian movements susceptible to reversible stop-and-goes due to the interactions of the receptors with stable anchoring proteins (acting as “diffusion traps”—mainly at synapses) and to the molecular crowding of nonspecific obstacles (the “pickets and fences”) (Figure 1). Therefore, the surface diffusion of neurotransmitter receptors is influenced by the protein and lipid composition of the receptor microenvironment, resulting in highly heterogeneous surface dynamics (Owen et al., 2009; Renner et al., 2009). Free receptor diffusion, with typical diffusion coefficient values of 0.1–1 μm^2^/s, is mainly observed at extrasynaptic compartments, whereas in specialized areas such as synapses, receptor diffusion coefficients can reach even three to four orders of magnitude smaller values, largely due to diffusion traps (Calamai et al., 2009; Petrini et al., 2009; Muir et al., 2010). Indeed, both at inhibitory and excitatory synapses, the interaction of neurotransmitter receptors with scaffold proteins represents the major cause of receptor transient corralling in the postsynaptic area, as in the case of GABA_A_R-gephyrin (Jacob et al., 2005), GlyR-gephyrin (Meier et al., 2001), AMPAR-PSD95-stargazin (Bats et al., 2007), mGluR-Homer (Sergé et al., 2002), D1 receptors-SAP102 (Thurner et al., 2014).

Receptor diffusion trapping at inhibitory synapses was first demonstrated in spinal cord neurons by the temporary stabilization and confinement of Glycine receptors (GlyRs) at synapses by the interaction of the receptor β subunit with gephyrin (Meier et al., 2001). Recently, Masson and colleagues have proposed that gephyrin acts as an energy trap for GlyRs, with a depth modulated by the biochemical properties of the receptor-gephyrin interaction domain (Masson et al., 2014). Gephyrin is also a scaffold protein for GABA_A_Rs. The binding of the α1 subunit intracellular TM3-4 loop with gephyrin E domain reduces the diffusion, mediates the accumulation and increases the dwell time of GABA_A_R at gephyrin-positive synaptic sites, thus concomitantly tuning the strength of synaptic inhibition (Mukherjee et al., 2011). In line with this, also the documented interaction of α2, α3, β2–3 and γ2 subunits of GABA_A_R with gephyrin sets the physical condition for the aforementioned diffusion trapping of GABA_A_R at synapses (Tretter et al., 2008; Maric et al., 2011; Mukherjee et al., 2011; Kowalczyk et al., 2013; Mou et al., 2013). It is worth noting that the major subunits composing extrasynaptic GABA_A_Rs lack binding sites for gephyrin, thus accounting for the exclusion of α1/4/6α5βγ receptors from synapses (Wu et al., 2012). During potentiation of tonic inhibition, when the surface delivery of α4 and α5 containing GABA_A_Rs is increased, the limited possibilities of these newly exocytosed receptors to be stabilized at synapses would favor their accumulation at extrasynaptic sites, despite they might transiently explore inhibitory synapses by lateral diffusion (Renner et al., 2012; Saliba et al., 2012; Bright and Smart, 2013; Abramian et al., 2014). Although, the α5 subunit of GABA_A_Rs has been mainly observed at extrasynaptic sites, some evidence indicates that it can also localize at GABAergic synapses (Brünig et al., 2002; Wu et al., 2012). It has been proposed that the synaptic localization of α5 subunits might be indirectly mediated by the anchoring of other synaptic GABA_A_R subunits assembled within the same receptor (Brünig et al., 2002; Wu et al., 2012; Gerrow and Triller, 2014). Recently, the lateral mobility and synaptic accumulation of α2 and α5 subunit-containing GABA_A_Rs has been reported to be oppositely modulated by GABA_B_ receptor, likely due to the competition between these two GABA_A_R subtypes for binding slots on synaptic scaffold proteins (Gerrow and Triller, 2014). Therefore, the preferential localization and diffusion trapping of distinct GABA_A_R subunits at synaptic or extrasynaptic compartments would be governed by the subunit assortment of GABA_A_Rs and by the affinity of receptor-scaffold interactions. In line with this, the diverse affinities of α1, α2 and α3 subunits for gephyrin can confer different diffusion properties to the mobility of synaptic GABA_A_Rs (Maric et al., 2011). Of note, it has been recently quantified that dimeric inhibitory receptor fragments bind dimeric gephyrin with a ~25-fold enhanced affinity compared to their monovalent counterparts (Maric et al., 2014). Considering that the typical synaptic αβγ GABA_A_Rs bear 4 potential gephyrin-binding domains (two on α and two on β subunits) (Tretter et al., 2008, 2011; Mukherjee et al., 2011; Kowalczyk et al., 2013) and that gephyrin oligomerization can range from dimers to dodecamers (Linsalata et al., 2014), the multivalency of receptor-scaffold interaction represents an additional key regulator of inhibitory receptor stabilization at synapses.

Phosphorylation events can further modulate the affinity of the receptor-scaffold interactions, resulting in changes in synaptic efficacy. For instance, a phosphomimetic mutation of Thr 347 on the gephyrin-interacting domain of the GABA_A_R α1 subunit reduces the affinity of GABA_A_R-gephyrin binding, decreases receptor trapping at synapses and depresses inhibitory synaptic transmission (Mukherjee et al., 2011). Similarly, a PKC-mediated phosphorylation of Ser 403 within the cytoplasmic domain of the β-subunit of the GlyR increases receptor lateral mobility at synapses by reducing the binding affinity between GlyR intracellular loop and gephyrin, thus contributing to the plasticity of inhibitory synapses (Specht et al., 2011). Also the phosphorylation of gephyrin can tune the strength of the receptor tethering (Zacchi et al., 2014). It has been demonstrated by mass spectrometry that gephyrin harbors 22 phosphorylation sites which can influence gephyrin folding and clustering (Herweg and Schwarz, 2012; Kuhse et al., 2012; Tyagarajan et al., 2013; Tyagarajan and Fritschy, 2014). In particular gephyrin phosphorylation of Ser 270 by GSK3β or CDK5 and of Ser 268 by ERK negatively regulates the clustering of gephyrin and GABA_A_Rs at synapses, threfore affecting inhibitory synaptic transmission (Kuhse et al., 2012; Tyagarajan et al., 2013; Tyagarajan and Fritschy, 2014). Furthermore, the conformational change of gephyrin induced by phosphorylation-dependent prolyl isomerase (Pin1) increases the stability of the scaffold lattice and the strength of GlyRs anchoring (Zita et al., 2007).

The regulation of receptor diffusion trapping at inhibitory synapses exhibits an additional level of complexity represented by the receptor-scaffold interactions occurring at extrasynaptic areas (Ehrensperger et al., 2007; Calamai et al., 2009). The dynamic equilibrium of synaptic and extrasynaptic receptor/scaffold interactions has been satisfactorily described by several computational models assuming that: (i) receptor-scaffold complexes can be formed outside and inside synapses; (ii) both preformed receptor-scaffold complexes and receptors alone can enter and leave the synapse; and (iii) the interaction of the receptors with the postsynaptic scaffold mainly accounts for receptor stabilization within the synapse (Ehrensperger et al., 2007; Calamai et al., 2009; Gerrow and Triller, 2010; Haselwandter et al., 2011; Figure 1, left inset). In this context, it has been additionally postulated that the size of synaptic clusters is maintained by a dynamic equilibrium between scaffold-scaffold aggregating forces and receptor-receptor repulsions (Haselwandter et al., 2011). Moreover, it cannot be excluded that other protein-protein interactions among molecular components of the synapse, including other structural proteins within the PSD, can contribute to receptor (and/or receptor-scaffold) trapping (Figure 1, left inset).

A more comprehensive picture of receptor diffusion trapping should also take into account endocytic zones (EZs) as specialized compartments where receptors mobility can be transiently reduced and confined, similarly to synapses. It has been demonstrated that GABA_A_R β3 and AMPAR GluA2 subunits can interact with AP2 (Lee et al., 2002; Kittler et al., 2008; Smith et al., 2012), leading to a reversible trapping of surface GABA_A_ and glutamate receptors at EZs, respectively (Petrini et al., 2009; Smith et al., 2012). Indeed, after a temporary retention at EZs, GABA_A_ and AMPA receptors recover free lateral diffusion upon exit from these specialized areas. Therefore, in addition to receptor removal from the surface, EZs contribute to the regulation of synaptic receptor number by transiently retaining the mobility of surface receptors.

## Activity-driven modulation of GABA_A_ receptor dynamics for the expression of synaptic plasticity

**Figure 2:**
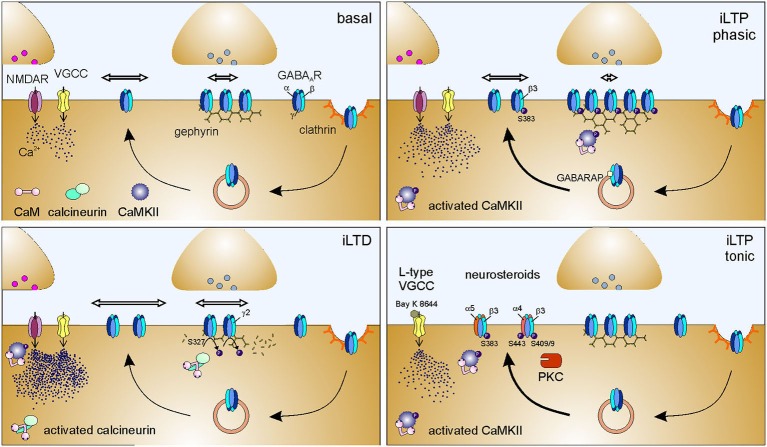
**Molecular mechanisms of postsynaptic plasticity of inhibitory synapses**. Basal: Simplified sketch of the molecular components of the inhibitory synapse in basal conditions. For schematization purposes, only the scaffold protein gephyrin and GABA_A_Rs are represented at the inhibitory PSD. Line arrows indicate GABA_A_R intracellular trafficking, namely exocytosis, clathrin-mediated endocytosis and recycling. Thicker line arrows indicate potentiated trafficking. Horizontal hollow arrows indicate surface GABA_A_R lateral mobility; the arrow length is proportional to receptor surface diffusion. Modifications of intracellular Ca^2+^ concentrations can be mediated by the activation of NMDA receptors (NMDARs) and/or voltage-gated calcium channels (VGCCs). The molecular changes occurring during iLTD and iLTP, schematized in the other panels, should be compared to the conditions represented here. iLTD: Postsynaptically-expressed inhibitory long-term depression (iLTD) is triggered by massive intracellular Ca^2+^ increase (mediated by NMDA receptors and/or VGCCs), that leads to the activation and recruitment of calcineurin to inhibitory synapses. Calcineurin mediates the dephosphorylation of Ser 327 on GABA_A_R γ2 subunit which in turn increases the lateral mobility of synaptic and extrasynaptic GABA_A_Rs, thus promoting the dispersion of synaptic receptors. Hence, inhibitory synapses exhibit a reduced number of GABA_A_Rs, resulting in decreased inhibitory synaptic strength. iLTD correlates with a reduction of gephyrin clustering at synapses (Lu et al., 2000; Wang et al., 2003a; Bannai et al., 2009; Muir et al., 2010; Niwa et al., 2012). iLTP phasic: Postsynaptically- expressed long-term potentiation of inhibitory synaptic currents (iLTP phasic) is elicited by NMDA-induced moderate increase of intracellular Ca^2+^ that recruits activated CaMKII to inhibitory synapse and promotes GABARAP-mediated GABA_A_R exocytosis. It cannot be excluded that VGCCs are contributing to the Ca^2+^ increase leading to iLTP. CaMKII phosphorylates at least Ser 383 on GABA_A_R β3 subunit, an event that enhances the accumulation of gephyrin at the postsynaptic level and selectively promotes the immobilization of synaptic GABA_A_Rs, while leaving the lateral diffusion of extrasynaptic receptors unaltered. As a result, inhibitory synapses are endowed with a larger number of GABA_A_Rs that accounts for increased inhibitory synaptic strength (Marsden et al., 2007; Petrini et al., 2014). iLTP tonic: Long-term potentiation of tonic inhibition (iLTP tonic) is mediated by the activation of L-type VGCCs (demonstrated by the sensitivity to dihydropyridine Bay K 8644, which stabilizes the channel open state). The consequent moderate increase of intracellular Ca^2+^ promotes the CaMKII-mediated phosphorylation of Ser 383 on GABA_A_R β3 subunit that in turn enhances the exocytosis of α5-containing GABA_A_Rs. This results in an increased number of surface α5-containing GABA_A_Rs, that, being predominantly extrasynaptic, potentiate tonic inhibitory currents (Saliba et al., 2012). When tonic iLTP is promoted by neurosteroids, the activation of PKC leads to the phosphorylation of Ser 443 on α4 subunit and on Ser 408/409 on β3 subunit of GABA_A_R. These events enhance the exocytosis and the membrane stability of α4 subunit-containing receptors, resulting in potentiated tonic currents (Abramian et al., 2010, 2014 but see also Bright and Smart, 2013).

Over the last decade, compelling evidence has documented that receptor lateral diffusion can be modulated in response to changes of neuronal activity (Figure 1, right inset). It has been reported that the impairment of synaptic activity by preventing action potential firing with the sodium channel blocker tetrodotoxin (TTX) significantly reduces the lateral diffusion of GABA_A_Rs in hippocampal cells (Bannai et al., 2009), although this effect has not been observed in spinal cord neurons (Lévi et al., 2008). In the hippocampus, a similar GABA_A_R immobilization was induced by blocking glutamatergic transmission with CNQX and APV (Bannai et al., 2009). Conversely, the lateral mobility of GABA_A_Rs increased upon induction of neuronal hyperactivity either by blocking potassium channels with 4-aminopyridine (4AP) or by dampening inhibition with GlyR and GABA_A_R antagonists (Bannai et al., 2009; Niwa et al., 2012), thus suggesting a positive correlation between GABA_A_R lateral diffusion and neuronal activity (Figure 1, right inset). The bidirectional modulation of receptor lateral mobility by neuronal activity provides a further control of receptor number at synapses during synaptic plasticity. It should be emphasized that this mechanism operates in the tens-of-milliseconds time range, being considerably faster that receptor recycling (Choquet and Triller, 2013). The role of receptor lateral mobility in the expression of long-term synaptic plasticity has been addressed in several studies at both glutamatergic and GABAergic synapses (Bannai et al., 2009; Makino and Malinow, 2009; Petrini et al., 2009, 2014; Muir et al., 2010). For instance, it has been recently demonstrated that the postsynaptic potentiation of inhibition, chemically induced in cultured hippocampal neurons by moderate NMDAR activation, relies on the enhanced accumulation and immobilization of surface GABA_A_Rs at synapses (Petrini et al., 2014; Figure 2). Such immobilization of synaptic GABA_A_Rs during iLTP has been explained by the promoted clustering of gephyrin at synapses. It has been proposed that the NMDA-induced moderate intracellular Ca^2+^ rise that triggers iLTP increases the phosphorylation of Ser 383 on GABA_A_R β3 subunit by CaMKII (Petrini et al., 2014). This event is crucial to promote the surface delivery of GABA_A_Rs and the recruitment of gephyrin to inhibitory synapses (Marsden et al., 2007; Petrini et al., 2014). Following the general rule mentioned above—concerning the influence of scaffold availability on receptor diffusion trapping—the increased clustering of gephyrin at the inhibitory PSD during iLTP promotes the corralling and prolongs the residence time of synaptic GABA_A_Rs, thus increasing inhibitory synaptic strength (Petrini et al., 2014). Of note, by selectively tracking the mobility of surface GABA_A_Rs before and after iLTP induction, Petrini et al. (2014) demonstrated that also preexisting surface GABA_A_Rs are recruited and immobilized at synapses during potentiation of inhibition. This observation describes an additional source of GABA_A_Rs accumulated at synapses during iLTP, besides the previously known promoted CaMKII-dependent exocytosis of GABA_A_Rs (Marsden et al., 2007; Figure 2).

It has been recently reported that an analogous mechanism based on postsynaptic modifications of GABAergic synapses underlies a form of potentiation of inhibition *in vivo*. Indeed, two independent studies have addressed the molecular determinants of iLTP observed *in vivo* in principal cells of the layer IV of rat visual cortex (Nahmani and Turrigiano, 2014; Petrini et al., 2014) after a brief protocol of monocular deprivation (MD) at the peak of the critical period (Maffei et al., 2006). With immunoelectron microscopy and confocal imaging, both studies have demonstrated that postsynaptic GABA_A_Rs are more enriched in MD animals expressing iLTP as compared to controls (Nahmani and Turrigiano, 2014; Petrini et al., 2014). Moreover, Petrini et al. (2014) have also documented an increase of gephyrin clusters immunoreactivity after the MD protocol. Overall those pieces of evidence indicate that a coordinated control of GABA_A_R and gephyrin dynamics could be a general mechanism underlying the postsynaptic expression of inhibitory synaptic potentiation *in vitro* and *in vivo*, at least in the rat visual cortex.

An opposite regulation of GABA_A_R and gephyrin with respect to that observed during iLTP has been described during iLTD induced by (i) trains of depolarizing stimuli; (ii) pharmacologically-induced increased excitatory neuronal activity (4AP); or (ii) strong NMDA receptor activation (Lu et al., 2000; Bannai et al., 2009; Muir et al., 2010; Niwa et al., 2012). In particular, at molecular level, the depression of synaptic inhibition relies on the recruitment of activated calcineurin at inhibitory synapses and on the declustering of gephyrin, along with the dispersal of synaptic GABA_A_Rs by augmented lateral diffusion (Bannai et al., 2009; Marsden et al., 2010; Muir et al., 2010; Figure 2). The increased lateral mobility of surface GABA_A_R observed during iLTD (induced by 4AP or NMDA) correlates with reduced GABA_A_R synaptic dwell time and increased confinement area through the calcineurin-mediated dephosphorylation of Ser 327 of the γ2 subunit (Marsden et al., 2010; Muir et al., 2010). However, the involvement of calcineurin in mediating gephyrin synaptic decrease during iLTD depends on the stimulus applied. Indeed, after 4AP stimulation, calcineurin increases GABA_A_R lateral mobility and reduces both GABA_A_R and gephyrin clusters, whereas after NMDA application it only controls GABA_A_R cluster size (Niwa et al., 2012). This suggests that the regulation of gephyrin clustering during NMDA-dependent iLTD does not depend on calcineurin activity. The data discussed so far indicate that moderate or high intracellular Ca^2+^ rise oppositely affects GABA_A_R surface dynamics leading to either iLTP or iLTD, respectively (Bannai et al., 2009; Marsden et al., 2010; Muir et al., 2010; Petrini et al., 2014). This might be explained in terms of the regulated recruitment of CaMKII at inhibitory synapses. In this concern, Marsden et al. (2010) have proposed that low Ca^2+^ recruits activated CaMKII at inhibitory synapses, while high Ca^2+^ leads to calcineurin localization at GABAergic synapses, where it prevents the accumulation of CaMKII (Figure 2). It is worth discussing that, the plasticity of excitatory synapses follows an opposite Ca^2+^ rule with respect to that of inhibitory synapses, as low and high Ca^2+^ trigger LTD and LTP, respectively (Lee et al., 2003; He et al., 2011). Therefore, the spatiotemporal dynamics of Ca^2+^ concentration can determine the bidirectional plasticity of both excitatory and inhibitory synapses. The coordination of these convergent Ca^2+^ signaling pathways is expected to be a main determinant for the fine control of the excitation/inhibition balance (E/I).

Besides the long-term synaptic plasticity, lateral diffusion has also been described to be crucial for the short-term plasticity at glutamatergic synapses. During rapid repetitive synapse activation, lateral diffusion allows desensitized AMPA receptors to leave the synapse and to be replaced with mobile extrasynaptic naïve receptors in tens of milliseconds, thus favoring the recovery from high-frequency synaptic depression (Heine et al., 2008). Those pieces of evidence suggest that extrasynaptic receptors constitute a reservoir pool of receptors which may exchange with desensitized synaptic receptors, thereby representing a gear for controlling the fidelity of synaptic transmission during high-frequency synaptic activation. In this regard, the kinetics of receptor desensitization would set the time window in which surface receptor dynamics can contribute to synaptic strength. At GABAergic synapses, the impact of surface GABA_A_R dynamics on short-term synaptic plasticity has not been addressed yet. Nevertheless, the fact that GABA_A_Rs desensitized state(s) can live for milliseconds-to-seconds periods (Jones and Westbrook, 1995; Petrini et al., 2011) suggests that desensitized GABA_A_Rs might exchange between synaptic and extrasynaptic compartments, providing the theoretical background for a multi-scaled temporal regulation of GABA_A_R dynamics on the fidelity of high-frequency inhibitory synaptic transmission. Future investigations will be required to clarify how GABA_A_R gating and lateral mobility cooperate to achieve the fine tuning of synaptic strength as a function of the frequency of synaptic activity.

## Dynamics of other synaptic players during inhibitory synaptic plasticity

In addition to neurotransmitter receptors, other proteins of the PSD represent key players of synaptic function, including scaffold and adhesion proteins, as well as structural elements, such as cytoskeleton and microtubules (Gordon-Weeks and Fournier, 2014; Tyagarajan and Fritschy, 2014). Noteworthy, synaptic scaffold proteins can laterally diffuse at submembrane level and even more importantly, their diffusive properties can be regulated in activity-dependent manner (Hanus et al., 2006; Sharma et al., 2006). Hence, the expression of synaptic plasticity implies dynamic and efficient adjustments not only of neurotransmitter receptor number at synapses, but of the whole postsynaptic structure and composition. For instance, the enrichment of synaptic gephyrin described in Petrini et al. (2014) as a key step for iLTP expression is initially sustained by the recruitment of extrasynaptic gephyrin to synapses. Of note, despite the increase of synaptic gephyrin is necessary for the potentiation of inhibition, gephyrin redistribution to synapses does not precede the accumulation of synaptic GABA_A_Rs. This evidence challenges the traditional notion that unbound receptors can be exclusively tethered at free docking slots available at synapse and it points towards the concept that the dynamics of scaffold molecules occurs in concert with that of neurotransmitter receptors both at synaptic and extrasynaptic compartments. The idea that during synaptic plasticity changes in scaffold dynamics precede neurotransmitter receptor rearrangements has been similarly challenged by Niwa and coworkers. The authors demonstrate that, during iLTD, the dispersal of synaptic GABA_A_R precede the reduction of gephyrin cluster size (Niwa et al., 2012). Despite, the role of scaffold dynamics in the reorganization occurring at the synapse during inhibitory synaptic plasticity starts to be unveiled with respect to gephyrin (Niwa et al., 2012; Petrini et al., 2014), future investigations of this issue should be broaden to include other anchoring/structural proteins of the inhibitory PSD.

Numerous pieces of evidence indicate a tight cross-regulation of many proteins of the inhibitory PSD. For instance, the impairment of collybistin dampens gephyrin and GABA_A_R clustering (Jedlicka et al., 2009; Poulopoulos et al., 2009), the lack of dystrophin or of gephyrin reduces the stabilization of GABA_A_R in a subset of synapses (Kneussel et al., 1999; Knuesel et al., 1999; Yu et al., 2007), but also the knock-out of GABA_A_Rs subunits prevents the correct clustering of gephyrin (Essrich et al., 1998; Schweizer et al., 2003; Studer et al., 2006). Altogether this suggests that, theoretically, all synaptic elements can influence the localization and dynamics the other synaptic molecules (Specht and Triller, 2008). In this framework, it has been demonstrated that the adhesion proteins β1 and β3 integrin influence GlyR dwell time and gephyrin exchange at synapses, leading to altered inhibitory synaptic strength in the spinal cord (Charrier et al., 2010).

The mobility of gephyrin molecules has been distinguished in a fast and low component, namely rapidly oscillations with sub-micrometric lateral motion around their initial position with diffusion coefficients 10–20 times slower than GABA_A_Rs and slow non-stochastic movements of entire gephyrin clusters over minutes-to-hours periods (Hanus et al., 2006; Maas et al., 2006; Calamai et al., 2009; Dobie and Craig, 2011; Kuriu et al., 2012). Both components of gephyrin dynamics are dependent on the presence of the cytoskeleton and microtubules (Hanus et al., 2006). The disruption of F-actin slows down rapid rearrangements and slower lateral displacements of gephyrin, whereas the impairment of microtubules only increases the slow lateral dynamics of gephyrin clusters (Hanus et al., 2006). Moreover, synaptic gephyrin can be additionally modulated by neuronal activity (Bausen et al., 2006; Hanus et al., 2006). This suggests that the reduction of rapid gephyrin dynamics observed during increased synaptic activity (Hanus et al., 2006) may depend on activity-dependent adjustments of the cytoskeleton and intracellular Ca^2+^ levels, likely influencing receptors stabilization at synapses (Wei et al., 2004). Another synaptic molecule reported to laterally diffuse in the neuronal membrane is the adhesion protein neuroligin1 (NLG1; Giannone et al., 2013). Despite NLG1 is mostly expressed at excitatory synapses, it has been also found at inhibitory synapses interacting with gephyrin (Levinson et al., 2005; Varley et al., 2011). The dynamic behavior of surface NLG1 is strongly reduced by presynaptic neurexin (Nrx). The binding to Nrx-1β favors the interaction of NLG1 with the intracellular excitatory scaffold and confers confined mobility to NLG1 (Giannone et al., 2013). Moreover, the phosphorylation state of Tyr 782 on NLG1 regulates the preferential binding of NLG1 to PSD95 or gephyrin, thus determining the different location of NLG1 at excitatory or inhibitory synapses (Giannone et al., 2013). Considering those data, it can be hypothesized that the activity-induced phosphorylation of NLG1 would potentially define the enrichment of NLG1 at excitatory or inhibitory synapses through the regulation of NLG1 lateral diffusion. Recent evidence has documented that also the KCC2 transporter explores the postsynaptic membrane by lateral diffusion and is transiently stabilized both at excitatory and inhibitory synapses (Chamma et al., 2013). Importantly, KCC2 surface dynamics is activity-regulated, as indicated by the higher lateral mobility and reduced dwell time of KCC2 observed upon increased network activity. This dispersal of KCC2 is dependent on the dephosphorylation of Ser 940 (Chamma et al., 2013). Of note, the dephosphorylation of Ser 940 additionally accounts for the reduced chloride export and diminished intensity of hyperpolarizing GABAergic inhibition induced by NMDA receptor activation (Lee et al., 2011), therefore suggesting that the activity-regulated dynamics of KCC2 can tune the strength of synaptic inhibition.

## Intrasynaptic nanoscaled receptor dynamics

The advent of super-resolution techniques and the progresses in EM tomography have allowed dissection of the structure and composition of the PSD at the single molecule level with nanometer resolution, revealing its subsynaptic clustered organization. Indeed, both at excitatory and inhibitory synapses, scaffold elements and receptors are arranged in nanostructures <100 nm wide (Chen et al., 2011; Fukata et al., 2013; MacGillavry et al., 2013; Nair et al., 2013; Specht et al., 2013). Although, as mentioned above, the postsynaptic scaffold undergoes constant molecular renewal and exhibits some dynamic rearrangements within the synapse, the subsynaptic scaffold nanodomains primarily represent stable hotspots for neurotransmitter receptor confinement (Sharma et al., 2006; Nair et al., 2013; Specht et al., 2013). Therefore, the idea of neurotransmitter receptors being highly mobile at extrasynaptic areas and stabilized at synapses by scaffold molecules requires a paradigm shift towards a more complex view in which the heterogeneity of the PSD composition and the nanodomains-based repartition of receptor mobility are taken into account (Gerrow and Triller, 2010; Choquet and Triller, 2013).

Importantly, such nanostructured organization of AMPA receptor dynamics has been reported to be activity-regulated, thus indicating that the exploring behavior of AMPA receptors at synapses is tuned by synaptic activity. Local activity confines AMPA receptor dynamics in nanometer-sized intrasynaptic areas and reduces the diffusive exchange between synaptic and extrasynaptic compartments. On the contrary, at inactive synapses, AMPA receptors fully explore the PSD and are less efficiently retained at synapses (Ehlers et al., 2007). Interestingly, mild glutamate receptor activation is sufficient to increase the intrasynaptic lateral mobility of AMPA receptors, as observed upon partial photobleaching of the PSD (Kerr and Blanpied, 2012). Most of the knowledge of the nanoscaled dynamics of synaptic components has been achieved at excitatory synapses with the benefit from the conceptual and technical advancement of different groups (Giannone et al., 2010; Hoze et al., 2012; Kerr and Blanpied, 2012; MacGillavry et al., 2013; Nair et al., 2013; Lu et al., 2014). Recently, superresolution techniques have been also exploited in the study of inhibitory synapses, describing for the first time the correspondence between the intrasynaptic spatial distribution of gephyrin and GlyR at spinal cord neurons (Specht et al., 2013). Furthermore, by assessing the stoichiometry of gephyrin-receptor binding, the authors studied the activity-dependence of gephyrin cluster occupancy by GlyR and GABA_A_R. They disclosed that, in spinal cord neurons, the blockade of spontaneous activity mostly affects the subset of inhibitory PSDs endowed with the largest GABA_A_R occupancy and lowest GlyR occupancy (Specht et al., 2013). This study lays the groundwork to deeply investigate in future years the nanoscopic rearrangements of the composition, distribution and dynamics of the synaptic components underlying the plasticity of inhibitory synapses. Furthermore, the precise location of GABA_A_R in the synaptic disc in relation to the position of the releasing site will be also crucial to determine the neurotransmitter concentration profile “seen” by postsynaptic receptors, an important determinant of synaptic strength (Barberis et al., 2011; Petrini et al., 2011).

## Conclusions

The correct functioning of the synapse relies on the balance between the stability of synaptic structures and the dynamics of its molecular components, a concept that is fundamental for basal synaptic transmission and for the activity-dependent tuning of synaptic strength. This Review highlights the importance of postsynaptic protein dynamics for the expression of plasticity at inhibitory synapses. A large body of evidence collected over the last 20 years has documented the highly coordinated, yet not fully elucidated, regulation of GABA_A_R intracellular trafficking (additionally modulated by receptor post-translational modifications), which controls surface receptor content to finally tune inhibitory synaptic efficacy. Remarkable technical and conceptual progresses achieved during the last decade have revealed that surface receptor lateral mobility is crucial to allow fast and persistent adjustments of ready-to-be-activated receptors at the synapse. Indeed, receptor lateral diffusion sustains (i) the constant renewal of synaptic receptors; and (ii) the activity-regulated dynamic redistribution of surface receptors to, within and from the synapse. The importance of postsynaptic protein dynamics in synaptic plasticity has been further reinforced when the notion of the postsynaptic scaffold has evolved from a “passive tether for synaptic receptors” towards a dynamic scenario in which receptor-scaffold interactions could be modulated over a wide range of time and intensities to ultimately regulate receptor diffusion trapping.

Despite our understanding of how activity-regulated protein dynamics contributes to inhibitory synaptic plasticity has significantly expanded, some of the underlying mechanisms remain fragmentary. In addition to the knowledge gaps highlighted throughout this Review, the comprehension of inhibitory postsynaptic plasticity will require to fully elucidate the influence of the molecular heterogeneity of GABAergic synapses on the dynamic remodeling of the synaptic molecular components. In addition to gephyrin, many other scaffold proteins composing the inhibitory PSD (eventually expressed in different isoforms and splice variants), should be analyzed during activity dependent synaptic reorganization. In this regard, the preferential binding of GABA_A_R subtypes to selected anchoring proteins would provide an additional level of complexity in the study of the modulation of receptor-scaffold interactions in basal conditions and during synaptic plasticity. For instance, in perisomatic synapses of CA1 pyramidal cells, in a subset of inhibitory synapses in cortical neurons, in cerebellar Purkinje neurons, the GABA_A_R α1 subunit is preferentially associated with the dystrophin-glycoprotein complex, whereas the α2 subunit better interacts with gephyrin (Panzanelli et al., 2011). Furthermore, it is still unclear whether the assortment and the synapse-specific localization of inhibitory postsynaptic scaffold proteins selectively modulate the expression of plasticity at subsets of synapses. This issue should also be contextualized to the rich diversity of GABAergic interneurons impinging on principal cells in specific compartments of the somato-dendritic axis (Klausberger and Somogyi, 2008).

The deeper investigation of the intracellular biochemical pathways activated during the plasticity of inhibitory synapses will also be fundamental to elucidate which mechanisms are shared with the plasticity of excitatory synapses for the future understanding of the coordination of activity-dependent adjustments of excitatory and inhibitory synaptic strength. The relevance of this issue lies in the functional crosstalk between excitatory and inhibitory synapses. That is, activity-dependent changes at excitatory and inhibitory synapses will not only result in altered E/I balance but also, at network level, they can be differently integrated to ultimately promote Hebbian plasticity or homeostatic stabilization of neuronal network activity (Vitureira and Goda, 2013).

Several lines of research have recently attempted to characterize *in vivo* inhibitory synaptic plasticity induced by environmental and chemical stimuli in different brain areas. For instance, multiple forms of potentiation and depression of GABAergic signaling have been described *in vivo* in response to changes of the animal sensory experience (e.g., ocular dominance, stress, fear acquisition and extinction) and to exposure to drugs (e.g., cocaine, ethanol, neurosteroids) (Maffei et al., 2006; Gonzalez et al., 2012; Li et al., 2012; Bocklisch et al., 2013; Inoue et al., 2013). Some of the molecular modifications and the cell-signaling pathways involved in the inhibitory synaptic plasticity *in vivo* start to be unveiled, as in the case of dynamic regulation of surface GABA_A_R and gephyrin levels during fear memory consolidation and after monocular deprivation (Chhatwal et al., 2005; Heldt and Ressler, 2007; Nahmani and Turrigiano, 2014; Petrini et al., 2014). However, there is increasing need to focus on protein dynamics *in vivo* for a more realistic description of the postsynaptic determinants underlying activity-dependent changes of inhibitory synaptic strength and their coordination. With the growing technological progress in neurophotonics, it is desirable to achieve *in vivo* the accuracy already obtained *in vitro* about the precise involvement of receptor and scaffold dynamics in the expression of inhibitory synaptic plasticity.

## Conflict of interest statement

The authors declare that the research was conducted in the absence of any commercial or financial relationships that could be construed as a potential conflict of interest.
